# Evaluation of five rapid diagnostic tests for detection of antibodies to hepatitis C virus (HCV): A step towards scale-up of HCV screening efforts in India

**DOI:** 10.1371/journal.pone.0210556

**Published:** 2019-01-17

**Authors:** Arati Mane, Jilian Sacks, Sadhya Sharma, Harpreet Singh, Alexandra Tejada-Strop, Saleem Kamili, Kartik Kacholia, Ritubhan Gautam, Madhuri Thakar, Radhey Shyam Gupta, Raman Gangakhedkar

**Affiliations:** 1 ICMR-National AIDS Research Institute, Pune, Maharashtra, India; 2 Clinton Health Access Initiative, New Delhi, India; 3 ART Plus Center, Government Medical College Amritsar, Punjab, India; 4 Division of Viral Hepatitis, Centers for Disease Control and Prevention, Atlanta, Georgia, United States of America; 5 National AIDS Control Organization, Ministry of Health and Family Welfare, Government of India, New Delhi, India; 6 Indian Council of Medical Research, Ministry of Health and Family Welfare, Government of India, New Delhi, India; Instituto Rene Rachou, BRAZIL

## Abstract

**Objectives:**

Hepatitis C virus (HCV) infection is a major contributor to morbidity and mortality worldwide. Early detection and curative treatment of HCV can reduce the risk of liver-related mortality and serve to prevent transmission of new infections. India is estimated to have about six million HCV infected individuals, most of whom are unaware of their infection status. Rapid diagnostic test kits (RDTs) could help identify HCV infected persons more expeditiously and thus availability of high performing, quality-assured RDTs is essential to scale-up HCV screening efforts. The present study was thus undertaken to evaluate the performance characteristics of five anti-HCV RDTs.

**Methods:**

Five anti-HCV RDTs (Alere Truline, Flaviscreen, Advanced Quality, SD Bioline and OraQuick) were evaluated using two panels of known anti-HCV positive and negative samples; one characterized from Indian patient samples (n = 360) and other obtained from the US Centers for Disease Control and Prevention (CDC), Atlanta (n = 100). Sensitivity, specificity, inter-observer agreement, test validity and operational characteristics of RDTs were assessed.

**Results:**

The combined sensitivities across both panels for Alere Truline, Flaviscreen, Advanced Quality, SD Bioline and OraQuick RDTs were 99.4% (95%CI-96.6%-99.9%), 86.2% (95%CI-79.8%-91.1%), 96.2% (95%CI-91.9%-98.6%), 99.4% (95%CI-96.6%-99.9%) and 99.4% (95%CI-96.6%-99.9%) respectively. The overall specificities across both panels for all RDTs were 99.7%. The inter-observer agreement was 100% for Alere Truline, SD Bioline and OraQuick, while it was 99.5% and 98.6% with Advanced Quality and Flavicheck respectively. Discordant results were significantly associated with human immunodeficiency virus (HIV) positivity for both Advanced Quality and Flavicheck (p<0.001).

**Conclusion:**

The present evaluation demonstrated that Alere Truline, SD Bioline and OraQuick RDTs had sensitivity and specificity in accordance with the acceptance criteria of the Drug Controller General, India, the national regulatory authority, had excellent inter-observer agreement and superior operational characteristics. Our findings suggest that certain HCV RDTs perform well and can be a useful tool in screening of HCV infections expeditiously.

## Introduction

Hepatitis C virus (HCV) infection is a major contributor to morbidity and mortality worldwide. It is estimated that globally 71 million people are living with chronic HCV infection, and about 400,000 succumb to this infection annually [1]. The burden of HCV infection is enormous in low and middle-income countries from the Southeast Asian region [2]. India alone is estimated to have about 6 million individuals living with chronic HCV infection, most of whom are unaware of their infection status [1, 3]. Chronic HCV infection is associated with long term complications such as liver fibrosis, cirrhosis and hepatocellular carcinoma [4]. HCV infection is primarily spread through exposure to contaminated blood, and rates of infection are particularly high among people who inject drugs (PWID) and men who have sex with men (MSM) [5, 6]. Since HCV and HIV share similar routes of transmission, co-infection rates are high and are associated with higher morbidity and mortality as well [7–9].

Early diagnosis and curative treatment of HCV infection can reduce the risk of liver-related morbidity and mortality and also serve to prevent transmission of new infections [10–13]. Furthermore, availability of efficacious, well-tolerated, relatively cheap, and easy to administer directly acting antivirals (DAAs), offer an opportunity to provide treatment and management using a public health model in India [14]. The first step to scaling up access to curative HCV treatment is to identify individuals who are chronically infected with HCV. Determination of current HCV infection requires screening for the presence of antibodies to HCV (anti-HCV) followed by confirmation of current infection either by using nucleic acid testing (NAT) for HCV RNA or an immunoassay (IA) for HCV core antigen wherever available [1]. Laboratory-based IAs, including automated platforms or manual enzyme immunoassays (EIAs), have proven accuracy in detection of HCV antibodies, however they require standard laboratory set up, skilled personnel, and have longer turn-around times (TAT), and necessitate extensive sample transport should screening be decentralized to sites without necessary infrastructure [15].

Rapid diagnostic tests (RDTs), eliminate the need for highly trained healthcare workers, sample transport, and provide fast TAT. Several commercial RDTs are available in the Indian market for the detection of HCV antibodies, however their performance characteristics have not been independently determined. Additionally, there are RDTs available globally that have not been approved for use in India by the Drug Controller General of India (DCGI). Evaluating their performance will help in decisions pertaining to approval of RDTs by DCGI. There have been previous reports of high rates of false negative anti-HCV rapid test results in HIV-HCV co-infected individuals [16, 17]; hence, evaluation of HCV RDTs in this group is essential.

The present study was conducted to evaluate the performance of five RDTs for detection of HCV antibodies. Data from this evaluation will provide valuable information for making decisions about scale-up of HCV screening in India as the government launches the National Program for the control of viral hepatitis.

## Methods

### Evaluation panels

Two panels were used in this evaluation, one sourced from patients in India and the other from the Division of Viral Hepatitis Laboratory, US Centers for Disease Control and Preventions, (US-CDC) Atlanta, GA (Table 1). The Indian panel was composed of a mix of both serum and plasma and includes representative samples from different geographical areas of the country, collected during years 2015–17 and stored at -70°C as part of sample repository at ICMR-National AIDS Research Institute (NARI). Samples from the co-infected (anti-HCV positive and HIV-positive samples) were prospectively collected in collaboration with the ART Plus Center, Amritsar, Punjab after obtaining written informed consent from donors. The CD4+ cell count and antiretroviral treatment data was obtained from the medical records.

The US-CDC, consisted of 100 anonymized well characterized plasma samples, previously tested for all HCV markers [18]. These plasma samples were collected from a US plasma donor center that were rejected due to anti-HCV-reactivity and/or HCV-RNA-positivity

**Table 1:** Description of the two specimen panels used for evaluation.

Anti-HCV				Total
**Indian panel**	**Anti-HIV**			**360**
	Positive		Negative	
Positive	N = 60 30 serum and 30 plasma		N = 60 40 serum and 20 plasma	120
Negative	N = 120 50 serum and 70 plasma		N = 120 100 serum and 20 plasma	240
**US-CDC panel**	**HCV RNA**			**100**
	Positive		Negative	
Positive	N = 25		N = 15	40
	18 = GT1a	2 = GT1b	NA	
	1 = GT2b	4 = GT3a		
Negative	N = 46		N = 16	60
	26 = GT1a	1 = GT1b	NA	
	10 = GT2b	9 = GT3a		

GT: genotype, NA: Not applicable

### Reference tests

The samples from the Indian panel were screened for anti-HCV by both Ortho HCV version 3.0 (Ortho Clinical Diagnostics, USA) and Murex anti-HCV version 4.0 (Diasorin, Italy) ELISA. All positive samples were tested by Bio Rad Western Blot to confirm antibody positivity as a supplemental test. A sample was considered positive when it was positive by both ELISA tests and Western Blot and negative when it was negative by both ELISA tests and Western Blot. For HIV status, the samples were tested by two ELISA Genetic Systems HIV-1/HIV-2 Plus O EIA (Bio Rad, USA) and HIVASE 1+2 ELISA (General Biologicals Corp, Taiwan) and three rapid tests, Alere Determine HIV 1/2 (Alere, Ireland), HIV Tri Dot (J Mitra Co Pvt Ltd, India) and HIV 1/2 Stat Pak Dipstick (Chembio Diagnostic Systems, USA). A sample was considered positive when it was positive by all five tests and confirmed by New LAV Blot Western blot (Bio Rad, USA), while negative when it was negative by all 5 tests. Discordant or indeterminate samples were not included in the panel. All assays were performed according to manufacturer instructions including criteria for analyte reactivity. Specimens in the US-CDC evaluation panel were tested for HCV markers as described previously, specifically using the Vitros 3600 CMIA platform (Ortho Clinical Diagnostics) for HCV serology [18]. All samples were plasma/serum; no whole blood specimens were analyzed.

### Anti-HCV rapid diagnostic test kits for evaluation

Five anti-HCV RDTs were evaluated: Alere Truline rapid test kit for HCV antibodies, Alere Medical Pvt Ltd, Gurgaon, India (Product code: 11304191030), Flaviscreen Plus HCV, Qualpro Diagnostics, Goa, India (Product code: 402170050), Advanced Quality Rapid Anti-HCV Test, Intec Products, Inc, Xiamen, China (Product code: ITP01151-TC40), SD Bioline HCV, Standard Diagnostics Inc, Republic of Korea (Product code: 02FK10) and OraQuick HCV rapid antibody test, OraSure Technologies, Bethlehem, PA, USA (Product code: 0006656483). The kits were stored at room temperature as recommended by the manufacturer. All are in vitro, qualitative, immune-chromatographic, single use, disposable chamber tests that provide visual results within 20 minutes for anti-HCV detection. Alere Truline, Flaviscreen and SD Bioline use antigens from structural (core) and nonstructural (NS3, NS4, and NS5) regions, while Advanced Quality kit uses the NS2 region antigens additionally. The OraQuick uses antigens from core and NS3 and NS4 regions.

### Evaluation procedures

The RDTs were performed as per the manufacturers’ instruction manuals. The procedure for testing and interpretation of the results was similar for all assays. An assay was interpreted as negative if a control line was present (regardless of intensity) with no corresponding test line. The appearance of a control line and a test line indicated a positive result. A missing or broken control line indicated an invalid result, regardless of presence of test line. Each specimen was tested by one laboratory technician and read by 2 independent laboratory staff in addition to the performer. A rapid assay result was classified as positive if at least 2 of the 3 observers interpreted the assay as positive.

### Statistical analysis

Data analyses were performed using SPSS 17.0 (SPSS, Chicago) software. Sensitivity and specificity with confidence intervals (CIs) for each RDT was calculated by comparing results obtained using the RDT to the reference result. Inter-observer agreement between the three technicians was measured by Fleiss’ kappa statistics. Invalid rate was calculated as the proportion of total samples tested in which the result was invalid out of the total number of samples tested. Each RDT was assessed for its operational characteristics by same three technicians. Tests were scored for clarity of kit instructions, technical ease of use and ease of result interpretation. Each of these characteristics was allotted marks out of 5, giving a maximum of 15. Responses to individual questions were analyzed to assign an overall score.

### Ethical considerations

The study was approved by Ethics Committee of ICMR-National AIDS Research Institute, Pune, India and the Chesapeake Institutional Review Board in the USA. Written informed consent was obtained from participants prior to sample collection.

## Results

### Performance of anti-HCV rapid diagnostic test kits

The sensitivity and specificity across both panels and per panel for the five HCV RDTs evaluated are shown in Table 2. The overall sensitivities of the RDTs ranged from 86.3% (95% confidence interval (CI), 79.9%-91.2%) to 99.4% (95% CI, 96.6%-99.9%). The Flaviscreen and Advanced Quality RDTs exhibited lower sensitivities. All five RDTs had specificities of 100% (95% CI, 98.8%-100%).

**Table 2:** Performance of anti-HCV rapid diagnostic test kits.

Anti-HCV rapid diagnostic test kits	Panel	Sensitivity (95% CI)	Specificity (95% CI)
Alere Truline (Product code: 11304191030)	Indian	100% (96.9%-100%)	100% (98.5%-100%)
	US-CDC	97.5% (86.5%-99.9%)	100% (94%-100%)
	Overall	99.4% (96.6%-99.9%)	100% (98.8%-100%)
Flaviscreen (Product code: 402170050)	Indian	88.3% (81.2%-93.5%)	100% (98.5%-100%)
	US-CDC	80% (64.4%-90.9%)	100% (94%-100%)
	Overall	86.3% (79.9%-91.2%)	100% (98.8%-100%)
Advanced Quality (Product code: ITP01151-TC40)	Indian	95.8% (90.5%-98.6%)	100% (98.5%-100%)
	US-CDC	97.5% (86.8%-99.9%)	100% (94%-100%)
	Overall	96.3% (91.9%-98.6%)	100% (98.8%-100%)
SD Bioline (Product code: 02FK10)	Indian	100% (96.9%-100%)	100% (98.5%-100%)
	US-CDC	97.4% (86.5%-99.9%)	100% (94%-100%)
	Overall	99.4% (96.6%-99.9%)	100% (98.8%-100%)
OraQuick (Product code: 0006656483)	Indian	100% (96.9%-100%)	100% (98.5%-100%)
	US-CDC	97.4% (86.5%-99.9%)	100% (94%-100%)
	Overall	99.4% (96.6%-99.9%)	100% (98.8%-100%)

95% CI: 95% confidence interval, US-CDC: US-Centers for Disease Control and Prevention

**Fig 1:**
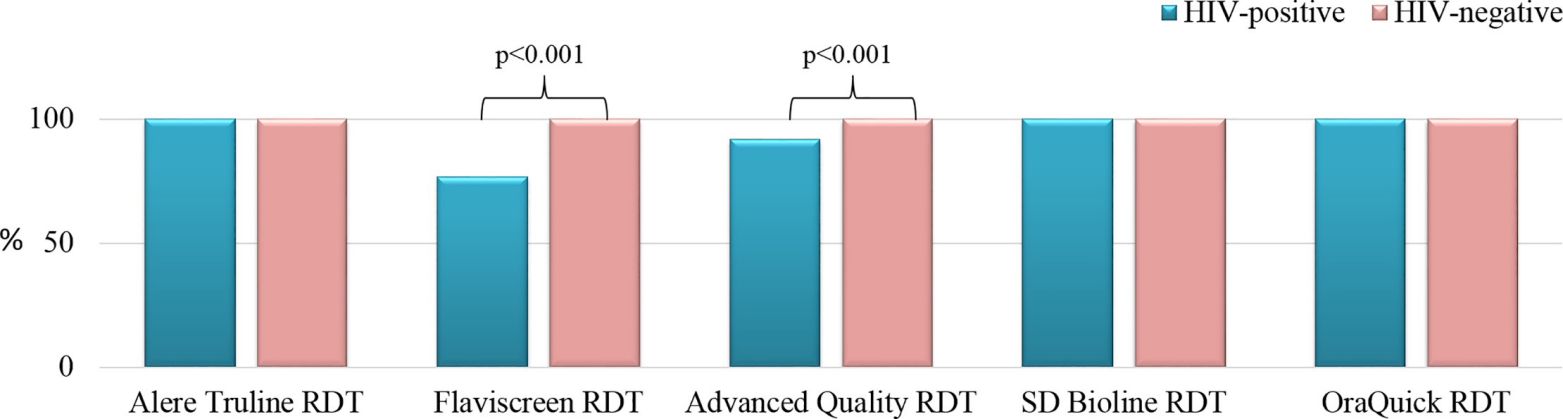
Sensitivities of anti-HCV rapid diagnostic test kits by HIV sero-status.

Using the Indian sourced panel, the sensitivity and specificity for the RDTs was evaluated according to HIV serostatus. We observed that Alere Truline, SD Bioline and OraQuick had 100% (95% CI, 94.0%-100%) sensitivities for both HIV-positive and for HIV-negative samples. The sensitivity of the Flaviscreen kit for HIV-positive samples was 76.7% (95% CI, 63.9%-86.6%) and for HIV-negative samples was 100% (95% CI, 94.0%-100%), while the sensitivity of Advanced Quality kit for HIV-positive samples was 91.7% (95% CI, 81.6%-97.2%) and for HIV-negative samples was 100% (95% CI, 94.0%-100%). There was a statistically significant difference between the sensitivities for HIV-positive and HIV-negative samples for both Flaviscreen and Advanced Quality RDTs (p<0.001) [Fig 1]. We did not observe any association between the discordant test results in the HIV-positive samples with CD4 cell count or antiretroviral treatment status. Using the US-CDC panel, we analyzed the influence of HCV viral load on the performance of RDTs and found no statistically significant difference between concordant (mean log HCV viral load: 5.1±1.2) and discordant (mean log HCV viral load: 4.9±3.5) RDT results, p = 0.45.

All five RDTs had 100% (95% CI, 96.9%-100%) specificity for both HIV-positive and for HIV-negative samples. False negative anti-HCV results were observed in 14/60 (23.3%) and 5/60 (8.3%) HIV-HCV co-infected samples with Flaviscreen and Advanced Quality RDTs respectively from the Indian Panel. HCV genotype data was available for all HCV RNA positive CDC samples, of which the same 2 samples (GT3a and GT1a) gave discordant results with Flaviscreen and Advanced Quality RDTs. There was one HCV seropositive sample in the US panel that was missed by all RDTs, which was HCV RNA negative.

### Inter-observer agreement, test validity and operational characteristics

The inter-observer agreement in the test results between three technicians was excellent for SD Bioline, Oraquick and Alere Truline HCV RDTs. The inter-observer agreement for the Advanced Quality HCV RDT was 0.993 and for Flaviscreen HCV RDT was 0.979 (Table 3).

**Table 3:** Inter-observer agreement for anti-HCV rapid diagnostic test kits.

Anti-HCV rapid diagnostic test kits	Agreement (%)	Kappa (95% CI)
Alere Truline	100%	1.00 (0.94–1.05)
Flaviscreen	98.6%	0.97 (0.93–1.03)
Advanced Quality	99.5%	0.99 (0.94–1.04)
SD Bioline	100%	1.00 (0.94–1.05)
OraQuick	100%	1.00 (0.94–1.05)

95% CI—95% confidence interval

No invalid test results were observed with any of the five RDTs evaluated. The scores for operational characteristics of the RDTs are summarized in Table 4. The OraQuick obtained the highest score (14/15) with a significant superiority on clarity of kit instruction and ease of result interpretation, while Flaviscreen scored lowest on technical ease of use and ease of result interpretation.

**Table 4:** Operational characteristics of anti-HCV rapid diagnostic test kits.

Operational characteristics	Top score	Mean scores				
		Alere Truline	Flaviscreen	Advanced Quality	SD Bioline	OraQuick
Clarity of kit instructions	5	5	4	4	4	5
Technical ease of use	4	4	2	3	4	4
Ease of result interpretation	5	4	2	4	4	5
Overall scores	14	13	8	11	12	14

## Discussion

This study investigated the diagnostic accuracy of five RDTs using two well-characterized serum/plasma panels, one an Indian and panel and other from US CDC. We observed that Alere Truline, SD Bioline and OraQuick had higher overall sensitivities as compared to Flaviscreen and Advanced Quality RDTs. These data are consistent with prior reports noting the good performance of the OraQuick and SD Bioline tests in other countries [15, 19–21]. To our knowledge, this is the first study to report the performance characteristics of Alere Truline, Intec Advanced Quality and Flaviscreen anti-HCV RDTs. The DCGI has laid acceptance criteria (sensitivity >99% and specificity ≥98%) for anti-HCV rapid immunodiagnostic kits in India and based on these standards the Alere Truline, SD Bioline and OraQuick RDTs met the acceptance criteria. The Advanced Quality and Flaviscreen kits had very good specificity, but exhibited relatively low sensitivity and thus do not meet the required standards. With the need to rapidly scale up screening having poor sensitivity and thus potential missing HCV-infected individuals would present a missed opportunity for the program.

It has been estimated that the prevalence of HCV in India is between 0.5 and 1.5 per cent, but the majority of individuals remain undiagnosed [14]. Though a national serosurvey has not yet been conducted, it is hypothesized that there is wide variation in state-to-state prevalence. The Indian state of Punjab, for example, is already known to have high HCV disease burden and has launched two public health programs the tackle the HCV epidemic accordingly [22–24]. Thus far, both state programs are catering to the latent demand of HCV treatment for few patients/risk groups who are already aware of their infection status, but there are plans to expand active screening and surveillance activities through community-based screening efforts in the near future. Furthermore, the Government of India has launched the National Program for Control of Viral Hepatitis and announced free treatment for hepatitis C [25]. Thus there is an urgent need to expand HCV diagnostics. RDTs could help identify the HCV infected persons more expeditiously; the rapid TAT will also assist to limit loss to follow-up and facilitate early linkage to treatment and care. As a step towards scale-up of HCV screening efforts in India, the present study was conducted to evaluate the diagnostic performance of five anti-HCV screening RDTs. To scale up access to HCV testing, integration of anti-HCV RDTs into established HIV testing services will be critical, including the use by organizations that are effective in reaching hard-to-reach or marginalized populations, such as PWID or MSM, who have not been tested for HCV, but are at higher risk of exposure. Therefore, it is critical that the performance of screening tests in the context of HIV co-infection be interrogated.

It has been reported that HIV-positive individuals may have impaired HCV antibody response [26–28]. Previous studies have reported false-negative results among HIV-positive individuals for HCV antibody detection, including with the OraQuick test [16, 17], which was not found for this test in our study. This could also reflect geographic differences in the origin of the samples. In the present study we observed false negative results in the HIV-HCV co-infected samples, using serum/plasma specimens, and consequently lower sensitivity for the Flaviscreen and Advanced Quality RDTs. No false negative results or statistically significant difference in the sensitivities with Alere Truline, SD Bioline and Oraquick RDTs were observed when samples from HIV-positive individuals were tested. These findings confirm that choice of appropriate HCV RDTs is essential, especially in the context of HIV co-infection.

Our study has a few limitations. All assays were performed in a reference laboratory by trained technicians. Hence the results may not be easily generalizable to the field setting where environmental conditions, sample type (whole blood) and expertise of technicians may vary. Though we analyzed the effect of HIV status on kit performance, there was an overall limited sample size, likewise other covariates like age, gender and the influence of other co-infections such as hepatitis B were not determined. As well, the virological profile of the Indian panel was not known, therefore influence of genotype or amount of virus cannot be accounted for. Lastly, both panels consisted of serum/plasma samples whereas the most likely and feasible sample type that would be in use in the field would be capillary (finger-stick) blood.

In summary, the present evaluation demonstrated that Alere Truline, SD Bioline and OraQuick RDTs had sensitivity and specificity in accordance with the acceptance criteria for anti-HCV RDTs as per the DCGI guidelines and had excellent inter-observer agreement and operational characteristics. The Flaviscreen and Advanced Quality kits demonstrated insufficient diagnostic accuracy, especially among HIV-infected individuals, suggesting that choice of appropriate HCV RDTs may be essential for use in the context of HIV-HCV co-infection.
